# Evaluation of Potential Clinical Surrogate Markers of a Trauma Induced Alteration of Clotting Factor Activities

**DOI:** 10.1155/2016/5614086

**Published:** 2016-06-28

**Authors:** Manuel Burggraf, Arzu Payas, Carsten Schoeneberg, Alexander Wegner, Max Daniel Kauther, Sven Lendemans

**Affiliations:** ^1^Department of Orthopedics and Emergency Surgery, University Hospital Essen, University of Duisburg-Essen, Hufelandstraße 55, 45147 Essen, Germany; ^2^Department of Emergency and Orthopedic Surgery, Alfried Krupp Hospital Steele, Hellweg 100, 45276 Essen, Germany

## Abstract

*Objective.* The aim of this study was to identify routinely available clinical surrogate markers for potential clotting factor alterations following multiple trauma.* Methods.* In 68 patients admitted directly from the scene of the accident, all soluble clotting factors were analyzed and clinical data was collected prospectively. Ten healthy subjects served as control group.* Results.* Patients showed reduced activities of clotting factors II, V, VII, and X and calcium levels (all *P* < 0.0001 to 0.01). Levels of hemoglobin and base deficit correlated moderately to highly with the activities of a number of clotting factors. Nonsurvivors and patients who needed preclinical intubation or hemostatic therapy showed significantly reduced factor activities at admission. In contrast, factor VIII activity was markedly elevated after injury in general (*P* < 0.0001), but reduced in nonsurvivors (*P* < 0.05).* Conclusions.* Multiple trauma causes an early reduction of the activities of nearly all soluble clotting factors in general. Initial hemoglobin and, with certain qualifications, base deficit levels demonstrated a potential value in detecting those underlying clotting factor deficiencies. Nevertheless, their role as triggers of a hemostatic therapy as well as the observed response of factor VIII to multiple trauma and also its potential prognostic value needs further evaluation.

## 1. Introduction

Multiple trauma is the most common cause of death in adolescence and young adulthood worldwide [1, 2]. While in deleterious traumatic brain injury (TBI) treatment options are usually limited, uncontrolled hemorrhage is deemed to be the most common preventable cause of death after trauma [3]. A considerable number of studies focused on the ideal administration of combinations of packed red blood cells, fresh frozen plasma, and/or hemostatic blood products. Yet, the ideal resuscitation protocol for traumatic hemorrhage still has to be found as no single intervention besides early application of tranexamic acid showed a clear superiority [4].

Currently, only few studies exist that systematically investigated clotting factor activities early after multiple injury. Rizoli et al. reported a critical deficiency of coagulation factor activities in 20% of severely injured patients at time of admission [5]. A subgroup analysis showed that the degree of hypoperfusion as reflected by levels of base deficit is associated with reduced activity in various coagulation factors [6]. Cohen et al., based on a subgroup analysis of data from the PROMMTT-study, demonstrated that clotting factor activities are reduced in coagulopathic major trauma patients receiving blood products [7]. Furthermore, a moderate reduction of clotting factor activities early after severe multiple trauma, irrespective of patient coagulation status, was shown [8]. However, all studies excluded a considerable number of patients from the final analysis according to certain patient characteristics (e.g., injury severity). We think that those results finally have to be proven for the majority of multiple trauma patients, as deleterious bleeding disorders are obviously more likely but not limited to certain patient subgroups. Moreover, in order to achieve clinical benefits from possible findings, reliable and easily available indicators of clotting factor impairments have to be found. Thus, the two primary objectives of this study were to generally analyze the clotting factor activities of multiple trauma patients immediately after admission to the trauma shock room and, secondly, to identify routinely available clinical parameters which could act as surrogate markers for the underlying clotting conditions.

## 2. Materials and Methods

### 2.1. Patients and Normal Donors

After sustaining multiple trauma, adult patients were enrolled into the study if admitted directly from the scene of the accident to the trauma bay at a Level 1 academic trauma center in Germany. Demographic data was collected prospectively. Ten healthy adult volunteers served as the control group. Patients with preexisting congenital or acquired coagulopathies and pregnant women were excluded from the study. The study was carried out in compliance with the Helsinki Declaration and was approved by the relevant local ethics committee (reference 12-5120-BO). Written informed consent was obtained from the participants. This study expands data from an original prospective study of severely injured patients by minor to moderate multiple trauma victims [8].

### 2.2. Blood Samples

Blood samples were drawn from a femoral artery directly after admission to the resuscitation room. Blood from healthy controls was drawn from a cubital vein. Blood samples were immediately transferred to the hospital laboratory. Besides standard blood tests, the activities of all remaining soluble clotting factors (FII, FV, FVII, FVIII, FIX, FX, FXI, FXII, and FXIII) were analyzed. The results are expressed as a percentage of standard activity. If the testing of clotting factor activity was not available right away (mainly due to off-duty hours), the specimens were cryostored at −70°C and analyzed the following weekday.

### 2.3. Statistical Analysis

IBM® SPSS® Statistics (Version 20) was used for statistical analysis and to compile the graphs. The* t*-test (age) and Fisher's exact test (gender) were used to determine demographic differences between patients and healthy volunteers. Differences between clotting factor activities of patients and controls were analyzed by using the Mann-Whitney* U*-test. Secondly, potential relationships between the clotting factor activities of traumatized patients and the patients' demographic and clinical data were investigated. For all scale and most ordinate variables Spearman's rank correlation, coefficient* rho* (*ρ*) was calculated to reveal possible relationships. Additionally, the 95%* confidence interval* (CI) was computed by bootstrapping using a bias-corrected and accelerated method based on 1000 bootstrap samples. The correlation was considered negligible for absolute values of *ρ* between 0.0 and 0.2, weak between 0.21 and 0.4, moderate between 0.41 and 0.7, strong between 0.71 and 0.9, and very strong between 0.91 and 1. When analyzing differences between distinct patient subgroups, the Mann-Whitney* U*-test was used. The subgroups were defined by gender, survival, preclinical fluid volume (< or ≥1500 mL), preclinical intubation, and administration of hemostatic agents (fibrinogen, tranexamic acid, prothrombin complex, and/or factor concentrates) within the hospital stay. Accordingly, a Kruskal-Wallis (one-way ANOVA by ranks) test was used to detect differences between three distinct trauma entities (blunt, penetrating, and isolated TBI). A *P* value smaller than 0.05 (2-tailed) was considered statistically significant for all tests. Demographic data is reported as means and standard deviation (SD), whereas results are reported as median values. All authors had access to primary clinical data.

## 3. Results

### 3.1. Demographic Data

68 multiple injured patients were enrolled into the final study. The mean age of 45 ± 17 years did not significantly differ from the control group (40 ± 9 years, *P* = 0.15). Overall, 82% of patients and 70% of healthy controls were male. Statistical analysis showed no significant difference in gender between both groups (*P* = 0.40). Patients showed a mean ISS of 24 ± 13 points and were admitted to the resuscitation room 63 ± 23 minutes after trauma. 59 patients suffered from blunt trauma, six from isolated TBI, and three from penetrating trauma. 13 patients showed a fatal outcome. Full demographic data is presented in Table 1.

### 3.2. Standard Coagulation Tests

**Figure 1:**
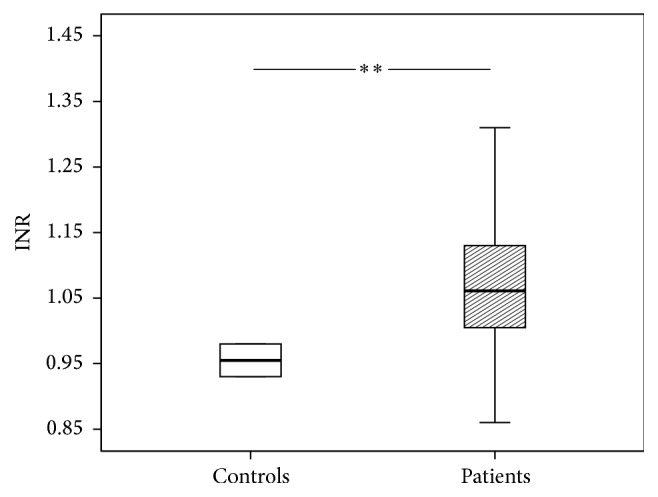
International Normalized Ratio (INR) of patients and control group. The results are presented by using boxplots. Bottom and top of the box indicate the 25th and 75th percentile, called interquartile range (IR). The median is represented by the horizontal bar within the box. Whiskers indicate spread (1.5 times IR). The Mann-Whitney* U*-test was performed with ^*∗∗*^ *P* = 0.001.

**Figure 2 fig2:**
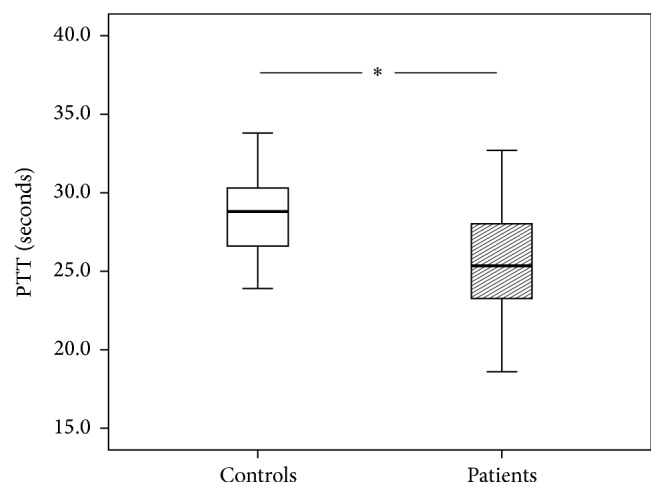
Partial Thromboplastin Time (PTT) of patients and control group. The results are presented by using boxplots. Bottom and top of the box indicate the 25th and 75th percentile, called interquartile range (IR). The median is represented by the horizontal bar within the box. Whiskers indicate spread (1.5 times IR). The Mann-Whitney* U*-test was performed with ^*∗*^ *P* < 0.05.

**Figure 3 fig3:**
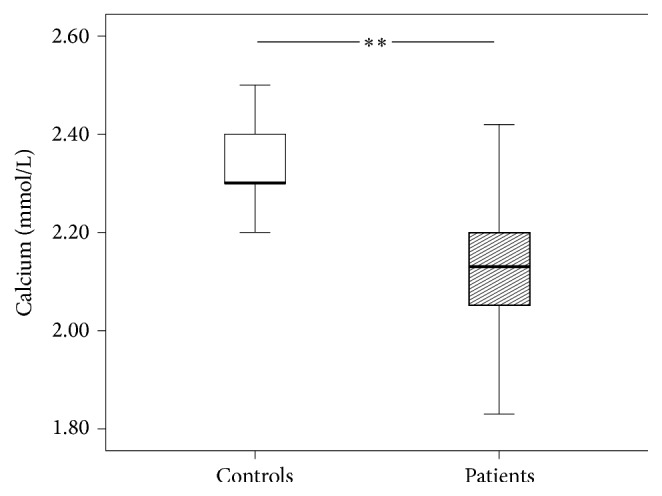
Calcium levels of patients and control group. The results are presented by using boxplots. Bottom and top of the box indicate the 25th and 75th percentile, called interquartile range (IR). The median is represented by the horizontal bar within the box. Whiskers indicate spread (1.5 times IR). The Mann-Whitney* U*-test was performed with ^*∗∗*^ *P* < 0.0001.

**Figure 4:**
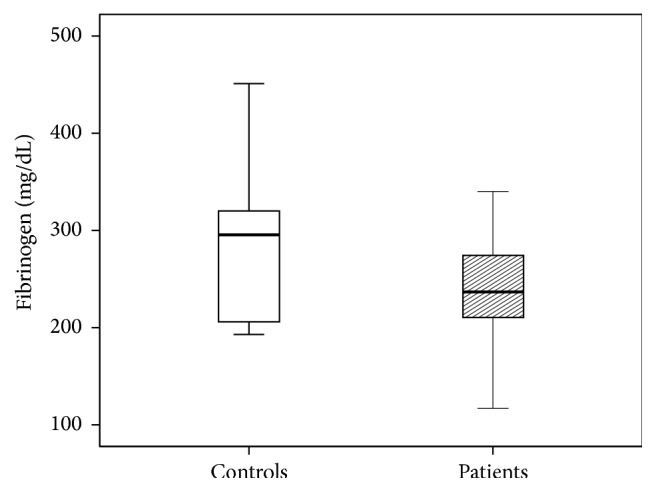
Fibrinogen levels of patients and control group. The results are presented by using boxplots. Bottom and top of the box indicate the 25th and 75th percentile, called interquartile range (IR). The median is represented by the horizontal bar within the box. Whiskers indicate spread (1.5 times IR). The Mann-Whitney* U*-test was performed to test for differences.

Routinely performed coagulation tests showed significant changes following multiple trauma (Figures 1 [Fig fig2] [Fig fig3]–4). The patients' International Normalized Ratio (INR) was elevated (1.06 versus 0.96, *P* = 0.001) whereas the Partial Thromboplastin Time (PTT) was shortened (25.3 versus 28.8 seconds, *P* < 0.05) compared to controls. Serum levels of calcium were reduced as well (2.13 versus 2.30 mmol/L, *P* < 0.0001). Fibrinogen showed a trend towards decreased levels. However, this reduction did not reach statistical significance (237 versus 296 mg/dL, *P* = 0.08).

### 3.3. Coagulation Factor Activity

**Figure 5:**
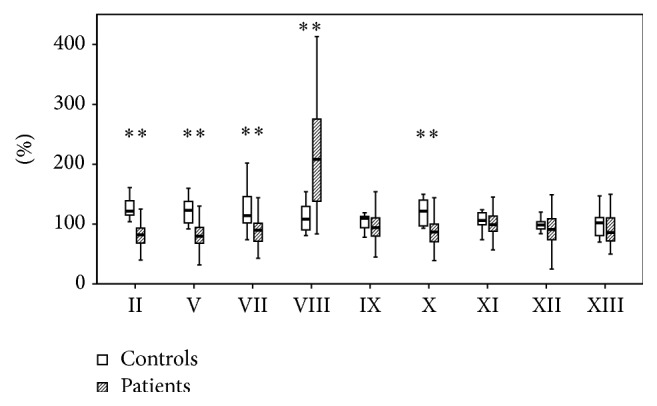
Clotting factor activities of patients and control group. The results are presented by using boxplots. Bottom and top of the box indicate the 25th and 75th percentile, called interquartile range (IR). The median is represented by the horizontal bar within the box. Whiskers indicate spread (1.5 times IR). The Mann-Whitney* U*-test was performed with ^*∗∗*^ *P* < 0.01.

Figure 5 shows the activities of the remaining soluble clotting factors. Final analysis showed significantly reduced activities of FII (82 versus 122%, *P* < 0.0001), FV (80 versus 123%, *P* < 0.0001), FVII (90 versus 114%, *P* < 0.01), and FX (88 versus 122%, *P* < 0.0001) whereas the activity of FVIII was elevated significantly (208 versus 109%, *P* < 0.0001). The remaining factors showed a trend towards reduced activity and serum levels, respectively.

### 3.4. Correlation Analysis

*Rho* and 95% confidence intervals for the relationship between clotting factors and the results of routine blood tests are given in Table 2. INR showed a moderate to strong negative correlation with fibrinogen levels and the activities of FII, FV, FVII, FIX, and FX. Furthermore, PTT showed a moderate to strong negative correlation with the activities of FII, FV, FVIII, FIX, FXI, and FXII. Levels of hemoglobin were also moderately related to a series of clotting factors (FII, FV, FIX, FX, FXI, FXII, and calcium). In addition, platelet counts (for FII and FXI) as well as BD (for FII, FV, and calcium) showed a few moderate correlations among the pairings. In contrast, levels of lactate did not show any relevant correlation at all.  Table 3 presents the relationships between clotting factors and selected demographic data. Final analysis showed moderate negative correlations between the injury severity score (ISS) and FIX and FXI. Moreover, the preclinical systolic blood pressure (SBP) correlated with the activities of FV, FIX, and FXII and the SBP at admission was related to FII, FIX, and FXI. Additionally, the time until admission to hospital showed a negative relationship with the activities of FIX and FXI. Finally, no relevant correlations between the levels and activities of clotting factors and the body temperature at admission or the age of the patients were found. All moderate to high correlations reached statistical significance (*P* < 0.0001 to 0.01).

### 3.5. Subgroup Analysis

Gender specific analysis did not show any significant differences in clotting factor activity between male and female patients. Analysis of the varying trauma entities blunt, isolated TBI, and penetrating showed significant differences among the three groups only for FXIII activity, which was significantly reduced after penetrating trauma (88 versus 69 versus 51%, *P* < 0.01).

**Figure 6:**
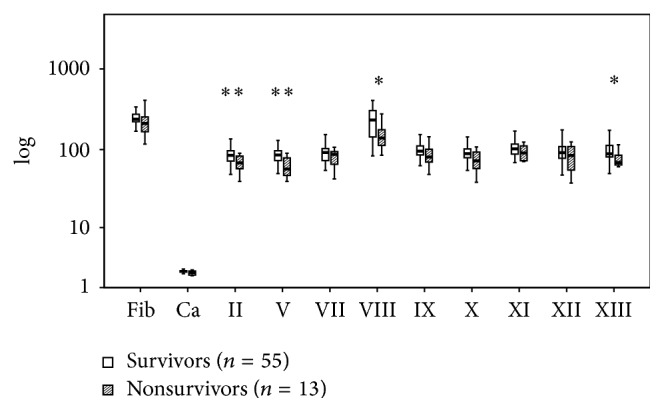
Clotting factor activities of survivors and nonsurvivors after multiple trauma. Fib: fibrinogen; Ca: calcium. The results are presented by using boxplots. Bottom and top of the box indicate the 25th and 75th percentile, called interquartile range (IR). The median is represented by the horizontal bar within the box. Whiskers indicate spread (1.5 times IR). Logarithmic ordinate for better depiction. The Mann-Whitney* U*-test was performed with ^*∗*^ *P* < 0.05 and ^*∗∗*^ *P* < 0.01 (survivors versus nonsurvivors).

**Figure 7 fig7:**
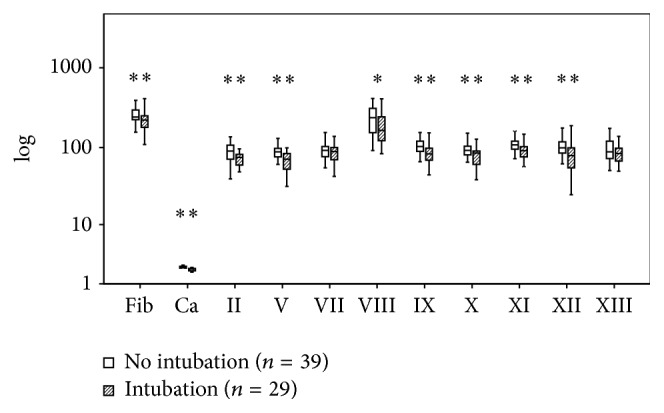
Clotting factor activities after multiple trauma and preclinical intubation. Fib: fibrinogen; Ca: calcium. The results are presented by using boxplots. Bottom and top of the box indicate the 25th and 75th percentile, called interquartile range (IR). The median is represented by the horizontal bar within the box. Whiskers indicate spread (1.5 times IR). Logarithmic ordinate for better depiction. The Mann-Whitney* U*-test was performed with ^*∗*^ *P* < 0.05 and ^*∗∗*^ *P* < 0.01 (intubation versus nonintubation).

**Figure 8 fig8:**
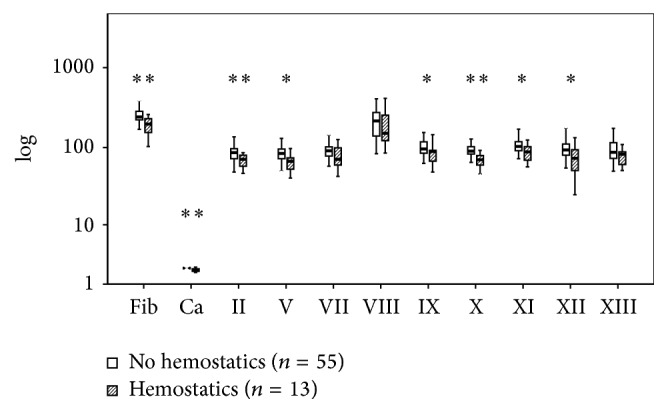
Clotting factor activities at admission after multiple trauma and the need for hemostatic therapy. Fib: fibrinogen; Ca: calcium. The results are presented by using boxplots. Bottom and top of the box indicate the 25th and 75th percentile, called interquartile range (IR). The median is represented by the horizontal bar within the box. Whiskers indicate spread (1.5 times IR). Logarithmic ordinate for better depiction. The Mann-Whitney* U*-test was performed with ^*∗*^ *P* < 0.05 and ^*∗∗*^ *P* < 0.01 (hemostatic versus nonhemostatic therapy within hospital stay).

**Figure 9:**
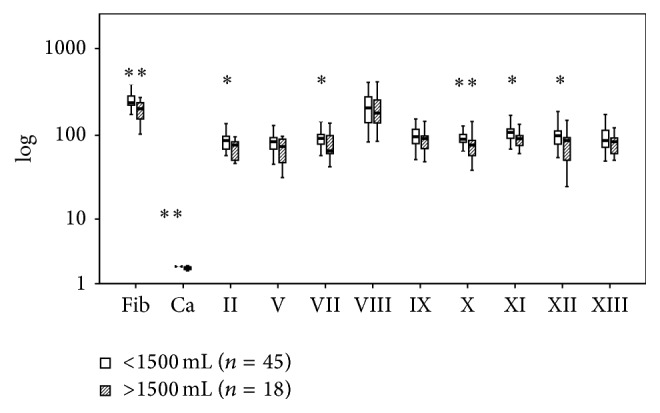
Clotting factor activities after multiple trauma according to amount of preclinical fluid resuscitation. Fib: fibrinogen; Ca: calcium. The results are presented by using boxplots. Bottom and top of the box indicate the 25th and 75th percentile, called interquartile range (IR). The median is represented by the horizontal bar within the box. Whiskers indicate spread (1.5 times IR). Logarithmic ordinate for better depiction. The Mann-Whitney* U*-test was performed with ^*∗*^ *P* < 0.05 and ^*∗∗*^ *P* < 0.01 (<1500 versus >1500 mL of preclinical fluid volume).

Figures 6 [Fig fig7] [Fig fig8]–9 present the differences related to survival, intubation, hemostatic therapy, and prehospital volume replacement. In contrast to survivors, nonsurvivors had significantly reduced activities of FII (66 versus 84%, *P* < 0.01), FV (54 versus 84%, *P* < 0.01), FVIII (141 versus 231%, *P* < 0.05), and FXIII (69 versus 88%, *P* < 0.05). If patients needed intubation prior to admission, the serum levels of fibrinogen (222 versus 242 mg/dL, *P* < 0.01) and calcium (2.06 versus 2.20 mmol/L, *P* < 0.01) as well as the activities of FII (75 versus 90%, *P* < 0.01), FV (71 versus 88%, *P* < 0.01), FVIII (164 versus 237%, *P* < 0.05), FIX (83 versus 103%, *P* < 0.01), FX (85 versus 92%, *P* < 0.01), FXI (91 versus 108%, *P* < 0.01), and FXII (79 versus 99%, *P* < 0.01) were reduced significantly. Patients who were in need for hemostatic therapy over the course had significantly reduced levels and activities of all soluble clotting factors but FVII (71 versus 91%, *P* = 0.06), FVIII (151 versus 216%, *P* = 0.35), and FXIII (73 versus 93%, *P* = 0.05). If the amount of prehospital volume replacement exceeded 1500 mL, levels of fibrinogen (203 versus 237 mg/dL, *P* < 0.01) and calcium (2.06 versus 2.16 mmol/L, *P* < 0.01) as well as activities of FII (77 versus 87%, *P* < 0.05), FVII (66 versus 92%, *P* < 0.05), FX (77 versus 90%, *P* < 0.01), FXI (91 versus 108%, *P* < 0.05), and FXII (87 versus 99%, *P* < 0.05) were decreased significantly.

## 4. Discussion

After multiple trauma, the activities of FII, FV, FVII, FX, and calcium levels are significantly reduced. In principle, this data should reflect the majority of multiple trauma patients, as no exclusion criteria in terms of preexisting treatment, ISS, or the subsequent clinical course were used. It must be said, though, that the reduction of clotting factor activities, although statistically significant, is rather small. None of the median activities falls short of the lower limits of its associated reference range (70%, as given by the laboratory). The results basically reflect findings in severely injured patients, but the measured activities are clearly less impaired if compared to highly selected patient cohorts (e.g., coagulopathic and/or in shock) [5–8]. Nevertheless, we think that the observed clotting factor activities raise the question whether simple administration of fresh frozen plasma is the best treatment option when attempting to correct suspected clotting factor deficiencies. There is growing experimental and clinical evidence that resuscitation fluids with higher ratios of clotting factors such as prothrombin complex concentrate might be beneficial in this context [9–11].

When looking for markers of impaired factor activities, the initial hemoglobin level appears to be a valuable indicator. This is in conjunction with the recent finding that hemoglobin acts very well as a trigger for initiating a coagulation therapy [12, 13]. As shock is considered to be the prime driver of a trauma induced coagulopathy (TIC) [14], we also assessed the prognostic value of different shock markers (SBP, BD, and lactate). The observed discrepancy in the disadvantage of lactate might partially explain why BD was a better predictor of traumatic coagulopathy in a retrospective analysis [15]. Hence, also various relationships with factor activities were found for BD (as for pre- and intrahospital SBP); strength of these relationships was generally of lesser extent and appeared rather randomly distributed. Nevertheless, the moderate correlation of BD with FII activity might be one explanation for the described relationship of initial base deficit values with subsequent transfusion requirements and also mortality in severely injured patients [16]. Thrombin, the proteolytically activated form of FII, is a crucial protease in the process of coagulation [17], and, indeed, in our study the activity of FII was significantly reduced in nonsurvivors. Excessive preclinical fluid resuscitation (>1500 mL) led to significantly reduced clotting factor activities and, not surprisingly, patients with reduced clotting factor activities needed hemostatic agents significantly more often. Although dilution is not any longer seen as the primary contributor to TIC, this underlines the need for a rational application of fluids not to aggravate coagulopathy and outcome as shown by Hussmann et al. [18, 19]. Finally, we found broadly reduced clotting factor activities after preclinical intubation. Need for intubation at the scene of an accident usually indicates higher injury severity and might lead to a prolonged time to admission. Hence, the origin of this relationship ultimately remains unclear, as neither ISS nor time to admission and also not body temperature demonstrated such a clear effect on the activity of soluble clotting factors, as it could be expected from the literature [15, 20].

Moreover, analysis showed a fair relationship of both INR and PTT, with a series of clotting factor activities early after multiple trauma. Yet, we cannot recommend those tests in general as both have well-known limitations [21]. In our study, multiple trauma led to a significant rise of the INR, reflecting a mild coagulopathic state of the patient cohort as a whole, whereas, in contrast, the observed PTT was significantly shortened. This fact might explain why some studies report higher rates of coagulopathic patients when taking INR (or Prothrombin Time) instead of PTT as criterion for coagulopathy [7, 22, 23]. We have previously hypothesized that the observed high levels of FVIII after severe trauma might influence the* in vitro* assay method for PTT measurement in terms of a reduced interval until clot formation [8]. Indeed, it was shown that high levels of FVIII, for example, in so-called heparin resistance, can lead to a shortened PTT [24–26]. This effect seems to be irrespective of the commercial reagent used for testing [27]. Importantly, the activity of FVIII in trauma patients seems to react rather contrary to the activities of the remaining factors [6–8]. Therefore, diagnosis of an overall coagulopathic state might be missed when focusing on PTT values in trauma patients. Interestingly, FVIII concentrations also strongly correlate with results of point-of-care viscoelastic tests [28]. Evidence from* in vitro* experimental heparin resistance due to high levels of FVIII indicates that elevation of FVIII activity could eventually bias viscoelastic tests as well [29]. Unfortunately, as use of viscoelastic tests is not yet routinely implemented in the trauma bay algorithm at our institution, we lack of sufficient data to evaluate this potentially relevant bias in a clinical setting.

The likeliest explanation of the posttraumatic elevation of FVIII activity is an acute phase reaction, although further mechanisms like liberation from injured tissues are conceivable, as FVIII is produced by a variety of cell types [30–32]. However, the (patho)physiological meaning of the elevated FVIII activity is currently unknown. When circulating in plasma, FVIII is bound to von Willebrand factor as a carrier protein. It is involved in the amplification and propagation of the coagulation process [17]. This effect is mediated via complex interactions, mainly based on specific binding and consecutive activation of platelets [17, 33–35]. A relative deficiency of FVIII, for example, as seen in nonsurvivors, might explain a recently described posttraumatic platelet dysfunction [36–39]. If true, this would indicate a true physiological role of the elevated FVIII activity. On the other hand, a posttraumatic FVIII elevation might also possess negative implications. So far, several studies showed that high FVIII levels are an independent risk factor for venous thrombosis [40]. Among other numerous risk factors, this could at least partially explain the high susceptibility of multiple trauma patients for a variety of thromboembolic complications [41].

This study comprises some important limitations. They include study design, specimen handling, confounding due to individualized pre- and intrahospital patient care, and generalizability in principle. Results of this study are based on a rather small study size from a single trauma center. Therefore, findings have to be questioned critically as larger study populations might have unveiled different results. This is especially true for the different trauma entities, as the incidence of blunt traumata outweighs penetrating injuries by far. Supposing that penetrating injuries may rather lead to hemorrhage compared to vast tissue traumatization, this difference might have a divergent impact on coagulation in general and clotting factor activity in particular. This potential influence of patient demographics was discussed previously, as it represents the medical reality in Germany but may lead to divergent results in populations with significantly higher rates of penetrating trauma [8, 42]. On the other hand, except for the studies of Cohen, Rizoli, and Jansen investigating 165, 110, and 71 selective patients, investigations based on large patient cohorts are still missing [5–7]. Specimen handling itself bears a risk due to different half-lives until analysis, but this is highly unlikely and the procedure is widely accepted [8]. Another important limitation of this study is the strict focus on the role of soluble clotting factors. However, hemostasis is not solely based on coagulation factors, but a rather dynamic process involving various cells until final clot formation [17]. Hence, we think that profound knowledge of clotting factor derangements early after trauma is inevitable to conceptualize well-founded strategies for hemostatic resuscitation.

## 5. Conclusions

Multiple trauma predominantly leads to an early reduction of activities of soluble clotting factors. However, this loss of activity is of rather small extent overall. In contrast, FVIII levels are markedly elevated after injury and this may influence standard PTT measurements. Initial hemoglobin and, with certain qualifications, base deficit levels demonstrated a potential value in detecting underlying clotting factor deficiencies, whereas neither ISS, body temperature, nor time to admission did show a clear relationship. Nevertheless, implementation of results into clinical pathways needs substantially larger study sizes and a multicenter approach.

## Figures and Tables

**Table 1:** Demographic data of patients. Data reported as mean ± SD or absolute value and percentage of the study group. Data acquisition upon arrival if not otherwise specified. *Volume replacement* refers to crystalloids and/or colloids. ISS: injury severity score; TBI: traumatic brain injury.

Patients (*n* = 68)		
Age	45 ± 17	Years
Gender	56 (82%)	Male
ISS	24 ± 13	Points
Mechanism of trauma	59 (87%)	Blunt
	3 (4%)	Penetrating
	6 (9%)	Isolated TBI
Mortality	13 (19%)	
Systolic blood pressure (preclinical)	131 ± 34	mmHg
Systolic blood pressure (admission)	132 ± 26	mmHg
Lactate	2.3 ± 1.8	mmol/L
Base deficit	−2.8 ± 3.8	mmol/L
Hemoglobin	12.4 ± 2.2	mg/dL
Temperature	35.8 ± 0.9	°C
Volume replacement (preclinical)	1048 ± 563	mL
Intubation (preclinical)	29 (43%)	
Time to admission	63 ± 23	Minutes

**Table 2:** Correlation of clotting factor activity and serum levels with routine blood tests. Table shows Spearman's rank correlation coefficient *rho* with 95% confidence interval (CI). INR: International Normalized Ratio; PTT: Partial Thromboplastin Time.

	Fibrinogen	Calcium	FII	FV	FVII	FVIII	FIX	FX	FXI	FXII	FXIII
INR	−.60 (−.78 to −.33)	−.32 (−.53 to −.06)	−.61 (−.77 to −.38)	−.67 (−.81 to −.47)	−.74 (−.87 to −.56)	−.12 (−.44 to .22)	−.43 (−.69 to −.13)	−.49 (−.70 to −.24)	−.35 (−.61 to −.06)	−.32 (−.55 to −.06)	−.37 (−.59 to −.12)
PTT	−.28 (−.50 to .00)	−.27 (−.51 to .03)	−.44 (−.64 to −.17)	−.45 (−.67 to −.17)	−.32 (−.54 to −.05)	−.72 (−.85 to −.53)	−.55 (−.74 to −.29)	−.36 (−.62 to −.07)	−.62 (−.77 to −.40)	−.52 (−.73 to −.25)	−.21 (−.45 to .05)
Platelet count	.31 (.04 to .54)	.29 (.02 to .54)	.42 (.17 to .62)	.15 (−.10 to .36)	.34 (.07 to .56)	.25 (−.04 to .51)	.31 (.05 to .52)	.38 (.13 to .57)	.41 (.15 to .62)	.16 (−.14 to .40)	.38 (.09 to .64)
Hemoglobin	.40 (.17 to .60)	.56 (.32 to .74)	.42 (.19 to .62)	.41 (.15 to .61)	.34 (.07 to .57)	.20 (−.07 to .47)	.52 (.32 to .69)	.59 (.39 to .75)	.43 (.21 to .62)	.36 (.09 to .60)	.41 (.13 to .63)
Lactate	−.29 (−.56 to .01)	−.31 (−.54 to −.03)	−.26 (−.48 to .03)	−.32 (−.55 to −.03)	−.15 (−.42 to .15)	−.07 (−.34 to .25)	−.07 (−.34 to .24)	−.07 (−.33 to .21)	−.06 (−.34 to .29)	−.17 (−.44 to .17)	−.31 (−.55 to −.02)
Base deficit	.37 (.09 to .60)	.58 (.33 to .75)	.46 (.22 to .65)	.61 (.41 to .76)	.33 (.05 to .57)	.15 (−.18 to .44)	.32 (.04 to .58)	.20 (−.03 to .43)	.27 (.01 to .49)	.36 (.09 to .58)	.24 (−.05 to .51)

**Table 3:** Correlation of clotting factor activity and serum levels with demographic data. Table shows Spearman's rank correlation coefficient *rho* with 95% confidence interval (CI). ISS: injury severity score; SBP: systolic blood pressure.

	Fibrinogen	Calcium	FII	FV	FVII	FVIII	FIX	FX	FXI	FXII	FXIII
ISS	−.36 (−.60 to −.05)	−.32 (−.59 to .02)	−.27 (−.55 to .10)	−.14 (−.46 to .24)	−.03 (−.33 to .27)	−.34 (−.64 to .03)	−.48 (−.75 to −.12)	−.18 (−.48 to .17)	−.48 (−.73 to −.11)	−.33 (−.61 to .03)	−.26 (−.56 to .06)
Age	.26 (−.02 to .52)	−.05 (−.29 to .20)	−.13 (−.39 to .14)	.09 (−.15 to .33)	.11 (−.16 to .37)	−.08 (−.33 to .18)	.09 (−.15 to .34)	−.13 (−.41 to .16)	−.03 (−.29 to .24)	.04 (−.22 to .30)	.04 (−.21 to .29)
SBP (preclinical)	.40 (.14 to .61)	.36 (.05 to .60)	.39 (.11 to .60)	.45 (.18 to .66)	.26 (−.01 to .48)	.22 (−.05 to .46)	.47 (.23 to .66)	.36 (.06 to .59)	.39 (.14 to .59)	.50 (.28 to .68)	.17 (−.11 to .44)
SBP (admission)	.35 (.02 to .62)	.39 (.09 to .62)	.41 (.11 to .65)	.40 (.15 to .60)	.24 (−.03 to .47)	.28 (−.01 to .50)	.43 (.12 to .68)	.27 (−.04 to .53)	.47 (.17 to .71)	.37 (.07 to .62)	.05 (−.30 to .34)
Temperature	.26 (−.05 to .52)	.39 (.05 to .64)	.21 (−.11 to .50)	.16 (−.15 to .45)	.00 (−.32 to .28)	.05 (−.26 to .35)	.15 (−.17 to .48)	.05 (−.28 to .36)	.23 (−.11 to .55)	.24 (−.05 to .52)	−.18 (−.47 to .14)
Time to admission	−.13 (−.44 to .17)	−.16 (−.45 to .12)	−.23 (−.48 to .09)	−.24 (−.49 to .07)	−.28 (−.57 to .08)	−.26 (−.54 to .00)	−.41 (−.64 to −.15)	−.26 (−.55 to .06)	−.48 (−.68 to −.22)	−.23 (−.51 to .10)	.07 (−.26 to .37)
